# The positive impact of honeybee activity on fennel crop production and sustainability

**DOI:** 10.1038/s41598-024-64283-2

**Published:** 2024-06-27

**Authors:** Mahmoud Abbas Ali, Ammar Al-Farga, M. A. Seddik

**Affiliations:** 1 Plant Protection Department, Faculty of Agriculture, South Valley University, Qena, Egypt; 2 Department of Biochemistry, Faculty of Sciences, University of Jeddah, Jeddah, Saudi Arabia; 3 Department of Bees Research, Agricultural Research Center, Plant Protection Research Institute, Giza, Egypt

**Keywords:** *Foeniculum vulgare*, Funnel, Agroecosystems, Honeybees, Crop production, Food crops, Food production, Fruit weight, Insect pollination, Reproductive success, Self-pollination, Egypt, Apis *mellifera*, Animal behaviour, Entomology, Zoology

## Abstract

This study investigates the ecological interaction between honeybees (*Apis mellifera*) and fennel (*Foeniculum vulgare*) plants, examining the mutual benefits of this relationship. Field experiments conducted in Egypt from December 2022 to May 2023 recorded diverse insect pollinators attracted to fennel flowers, especially honeybees. Assessing honeybee colonies near fennel fields showed improvements in sealed brood (357.5–772.5 cells), unsealed brood (176.3–343.8 cells), pollen collection (53.25–257.5 units), honey accumulation (257.5–877.5 units), and colony strength (7.75–10) over three weeks. Fennel exposure explained 88–99% of variability in foraging metrics. Comparing open versus self-pollinated fennel revealed enhanced attributes with bee pollination, including higher flower age (25.67 vs 19.67 days), more seeds per umbel (121.3 vs 95.33), bigger seeds (6.533 vs 4.400 mm), heavier seeds (0.510 vs 0.237 g/100 seeds), and increased fruit weight per umbel (0.619 vs 0.226 g). Natural variation in seed color and shape also occurred. The outcomes demonstrate the integral role of honeybees in fennel agroecosystems through efficient pollination services that improve crop productivity and quality. Fennel provides abundant nutritional resources that bolster honeybee colony health. This research elucidates the symbiotic bee-fennel relationship, underscoring mutualistic benefits and the importance of ecological conservation for sustainable agriculture.

## Introduction

Sweet fennel (*Foeniculum vulgare*) is a plant belonging to the Apiaceae family. It is a short-lived perennial native to southern Europe and is often grown as an annual in cooler climates^1^. There are two commercially important varieties of sweet fennel: *Foeniculum vulgare* var. dulce and *Foeniculum vulgare* var. vulgare^2^. Sweet fennel is widely distributed in Africa and Asia, known for its potential as a natural and biological preservative in food science^3^. Fennel is native to Southern Europe and the Mediterranean region and is cultivated throughout the temperate and subtropical regions of the world^4^ Fennel seeds are widely used for culinary purposes and have several uses in the pharmaceutical industry as well ^5^. Fennel contains various phytoconstituents, including flavonoids and phenolic compounds, which contribute to its medicinal properties ^6^. It is also used as a flavoring agent in food products and has traditional uses as an antispasmodic, diuretic, and antioxidant^7^. It is commonly used in Egyptian cuisine and traditional medicine for its various health benefits^8^.

Sweet fennel attracts a diverse assemblage of beneficial insects, insects play a significant role as pollinators of fennel. They are essential for achieving good returns in fennel seed crop^9^. Various insect species, including honeybee (*A. mellifera*) bees, butterflies, and flies, visit fennel flowers for pollination^10,11^, lacewings beetles bumble bees ants social wasps mud daubers, tachinid flies, and hoverflies^12^, and the bee species, including *Apis florea*, *A. cerana*, *A. mellifera*, and *A. dorsata* were the most frequent visitors to fennel flowers^13^. The activity of different bee species on fennel varied with different abiotic factors, such as bright sunshine hours, temperature, and relative humidity^14^. Which the fennel is a summer-blooming perennial that produces flowers attractive to a broad range of pollinators^15^.

Fennel nectar and pollen have an impact on bee broods. Honeybee (*A. mellifera*) collect and store pollen in colonies to ensure a strong worker bee population and brood production^16^. The presence of brood stimulates pollen foraging in honeybee (*A. mellifera*), particularly in high pollen-hoarding strains^17^. Pollen is an essential food source for bee larvae, providing them with protein, lipids, vitamins, and minerals^18^. Phytochemicals found in nectar, honey, and pollen can affect bee infection and survival. Consumption of certain phytochemicals can increase antimicrobial peptide expression in honeybee (*A. mellifera*) and reduce levels of deformed wing virus (DWV)^19^.

Insect pollinators have a significant impact on the quantity and quality of crop seed production. Different insect pollinators, such as honey bees and blowflies, have varying effectiveness in pollinating crops flowers, with honey bees being more effective^20^. The presence of insect pollinators, such as honey bees, leads to higher seed yields and better germination rates compared to when inflorescences are caged or self-pollinated^21^. The presence of insect pollinators during the flowering period of fennel leads to higher seed number and seed weight per umbel^9^. In addition, seeds from flowers that were freely pollinated by insects showed higher germination capacity compared to those isolated from insect visitors^21^. It also leading to higher seed set ratios and increased seed weights in plants such as sunflowers and rapeseed^22,23^.

However, the specific impact of fennel nectar and pollen on bee brood was not mentioned in the previous works. This study aims to investigate the influence of fennel nectar and pollen on bee foraging broods, focusing on delineating the precise effects of fennel nectar and pollen on bee brood development, and the impact of *honeybee (A. mellifera)* on some fennel seed properties.

## Materials and methods

### Study site

The research was conducted in a dedicated cultivation area of fennel (*Foeniculum vulgare*) located in Masakine, Qena, Egypt, precisely at 43° 13′ 27.55″ N, 5° 28′ 2.92″ E. The study spanned from 2022 to 2023, aligning with the fennel crop season.

### Experimental design

A Completely Randomized Design (CRD) was employed for this field experiment, carried out in a natural fennel habitat with abundant flowering fennel plants. The study involved two experimental groups: a control group without honeybee pollination and an experimental group with honeybee exposure. The selected location had minimal pesticide use, ensuring a favorable environment for honeybee activity.

### Insect sampling and identification

Insect sampling was performed at the study site during December 2022 and May 2023. Insects were collected using hand nets and photographed during the peak flowering stage of *F. vulgare*. The insects visiting the flowering umbels for pollen and nectar were collected by steady pace walking and sampling from random flowering fennel umbels along the transect. The insects were identified to morphotype levels, including bees, social wasps, and others.

### Pollination assessment

Various parameters were monitored in both experimental groups, including flower density, nectar production, honeybee visitation frequency, behavior, and duration on fennel flowers. Specific measures such as flowering onset and duration, the number of flowers per plant, pollen transfer efficiency (quantified through pollen load on honeybee bodies), seed set, and seed viability were meticulously recorded and analyzed.

#### Honeybee colony activity

##### Outgoing bees

The total number of outgoing bees from three colonies of F1 Carniolan bees, each headed by sister queens of the same age, were counted within 3 min per count per day using a stopwatch. The mean number of foragers per day was sufficient to study foraging activity^24^.

##### Ingoing bees

The number of honeybee workers entering their hives with and without pollen were counted at intervals over three minutes using a stopwatch.

##### Brood rearing activity

Four F1 Carniolan honeybee colonies, each with new queens, were used to measure brood rearing activity (sealed brood area, unsealed brood, pollen, and honey) in comparison with the blossoming period of field crops. Sealed brood area measurement was conducted at 12-day intervals using a frame divided into square inches by wire mesh, following the method of El-Shakaa^25^.

#### Seed set

To examine the impact of pollinator visitation on fennel plant pollination, plants were covered with organza cloth to exclude pollinators, while non-caged groups served as controls. Each plant was assessed for the number of total flowers, seed weight, seed color, and seed shape.

#### Compliance with regulations

All plant material utilization in this research strictly adhered to applicable local, regional, national, and international regulations. All methodologies employed were conducted in full compliance with relevant guidelines, regulations, and legislation.

#### Data analysis

Statistical analysis was performed using GraphPad Prism (version 9) software. Independent samples t-tests compared morphological attributes (length, weight, seed count) between bee-pollinated and non-pollinated controls. Additional t-tests were used to determine differences in inflorescence age and flower/seed number. One-way ANOVA tests evaluated variations across temporal intervals related to bee activities, including honey, nectar, and pollen collection behaviors, as well as metrics such as bee ingress/egress frequency, individual flower visits per minute, and brood cell distribution within hives. ANOVA also assessed honey and pollen yields and colony strength across experimental dates. Shapiro–Wilk tests ensured parametric test assumptions were met, and Tukey’s multiple comparisons tests identified specific inter-group differences. This rigorous quantitative approach systematically analyzed experimental observations, revealing meaningful patterns in the influence of bee pollination on key fennel plant and hive parameters.

Our utilization of plant material in this research strictly adheres to all applicable local, regional, national, and international regulations.

Therefore, we confirm that all methodologies employed in this study were conducted in full accordance with the relevant guidelines, regulations, and legislation.

### Data analysis

The statistical analysis was conducted using GraphPad Prism (version 9) software. An independent samples t-test was performed to compare morphological attributes (length, weight, seed count) between plants subjected to bee pollination versus non-pollinated controls. Additionally, t-tests determined if differences existed in inflorescence age and flower/seed number. Furthermore, one-way ANOVA tests evaluated variations across temporal intervals related to bee activities. These included measurements of honey, nectar, and pollen collection behaviors, as well as metrics such as bee ingress/egress frequency from hives, individual flower Visits/min, and brood cell distribution within hives. ANOVA also assessed honey and pollen yields and colony Strength between experimental dates. All response variables were first tested for normality using Shapiro–Wilk tests to ensure parametric test assumptions were met. Where significant, Tukey’s multiple comparisons tests identified specific inter-group disparities. This rigorous quantitative approach systematically analyzed experimental observations and revealed meaningful patterns in the influence of bee pollination on key fennel plants and hive parameters. Precisely executed statistical tests validated the described biological impacts.

### Ethical considerations and permissions

The study adheres to ethical guidelines for the treatment of honeybee colonies and the handling of bees. As the author of the manuscript, we affirm that all necessary permissions and licenses for the collection of plant and seed specimens were duly obtained in accordance with applicable regulations and ethical guidelines.

## Results

### Diversity of insect visitors on fennel

Throughout the study period, a wide variety of insects were documented as they visited the blooming umbels of the fennel. The collected data revealed a total of six distinct species, spanning five different orders, seven genera, and six families (Table 1). The observations unveiled diverse insect groups linked with fennel in the Qena region. Notably, these recorded pollinators include a range of bee species, such as honeybees, Carpenter bees, and parasitic wasps. Furthermore, the recorded observations in Table 1 reveal the presence of other insect groups contributing to fennel pollination. Hoverflies, beetles, seed bugs, and the pea blue were documented as frequent visitors, in association with fennel.

**Table 1:** Insect diversity associated with fennel flowers.

S. N	English name	Scientific name	Family	Order
1.	Honeybee	*Apis mellifera* L	Apidae	Hymenoptera
2.	Carpenter bee	*Xylocopa pubescens*	Apidae	Hymenoptera
3.	The Oriental hornet	*Vespa orientalis*	Vespidae	Hymenoptera
4.	Hoverfly	*Kristalina’s tabanoides*	Syrphidae	Diptera
5.	Variegated ladybug	*hippodamia variegata*	Coccinellidae	Coleoptera
6.	Seed bugs	*Spilostethus pandurus*	Lygaeidae	Hemiptera
7.	The pea blue	*Lampides boeticus*	Lycaenidae	lepidoptera

### Impact of fennel on bees’ activities

#### Foraging

##### Number of umbels visited/min

**Figure 1:**
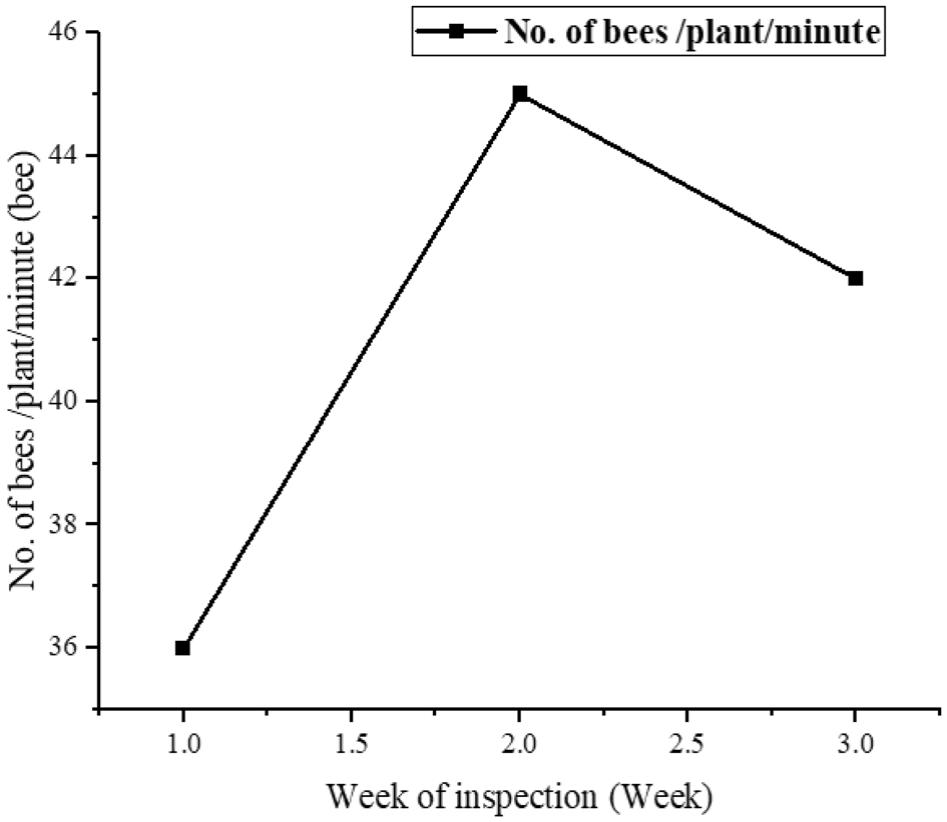
Relationship between fennel plant exposure and bee colony parameters.

The presentation (Table 2) reveals variations in bee foraging behavior on fennel flowers, specifically in terms of the number of umbels visited per minute. The data indicates an increasing trend in bee activity over the three inspection weeks, with bees visiting an average of 36 ± 2.309 umbels per minute during the first week, 42 ± 2.667 umbels per minute during the second week and reaching a peak of 48 ± 3.464 umbels per minute during the third week (Fig. 1). This suggests an escalation in foraging efficiency and activity as the week progresses.

**Table 2:** Bee foraging behavior variation on fennel flowers over inspection weeks.

Bees visiting	Visited (min)	Rate of chances
Frist week	36 ± 2.31	–
Second week	45 ± 2.667	6.358
Third week	42 ± 3.464	6.797
R squared (eta squared)	0.4778	–
p-value (95%)	0.1424	–
F value	2.745	–

One notable bee species, *Apis mellifera*, consistently demonstrates an elevated level of foraging activity throughout all inspection weeks. With a range of 36–48 umbels visited per minute, *Apis mellifera* exhibits remarkable efficiency in pollination, making it a significant contributor to the pollination process of fennel.

The statistical analysis supports these findings, with an R-squared value of 0.4778 indicating that 47.78% of the variation in umbels visited can be attributed to the different inspection weeks. However, the p-value of 0.1424 (at the 95% confidence level) suggests that the observed differences in bee activity across the inspection weeks are not statistically significant. The F value of 2.745 further supports this, indicating that the variations in umbels visited are due to random fluctuations rather than meaningful differences.

##### Fennel plant’s impact on bee foraging and activity

Data in Table 3 presented here explores the relationship between fennel plant exposure and bee foraging behavior and activity over a 3-week period.

**Table 3:** Effect of fennel plant exposure on honeybee foraging behavior and activity.

Foraging	Mean no. of bees					
	Bee in the first week	Bee in the second week	Out third week	R squared (eta squared)	p-value (95%)	F_0.05_
In	60.00 ± 1.95	86.50 ± 2.217	89.00 ± 3.342	0.8963	< 0.0001	38.90
Out	69.25 ± 2.287^c^	92.50 ± 2.598^b^	95.00 ± 2.483^a^	0.8811	< 0.0001	33.34
Pollen	13.25 ± 1.315	19.50 ± 1.848	15.75 ± 1.250	0.4958	< 0.0459	4.425
Honey	46.75 ± 0.7500	67.00 ± 2.972	73.25 ± 2.136	0.9016	< 0.0001	41.24

Means followed by the same letter within each row are not significantly different from each other at the 5% level (p>0.00). Different letters (a, b, c) indicate statistically significant differences between the weeks.

**Figure 2:**
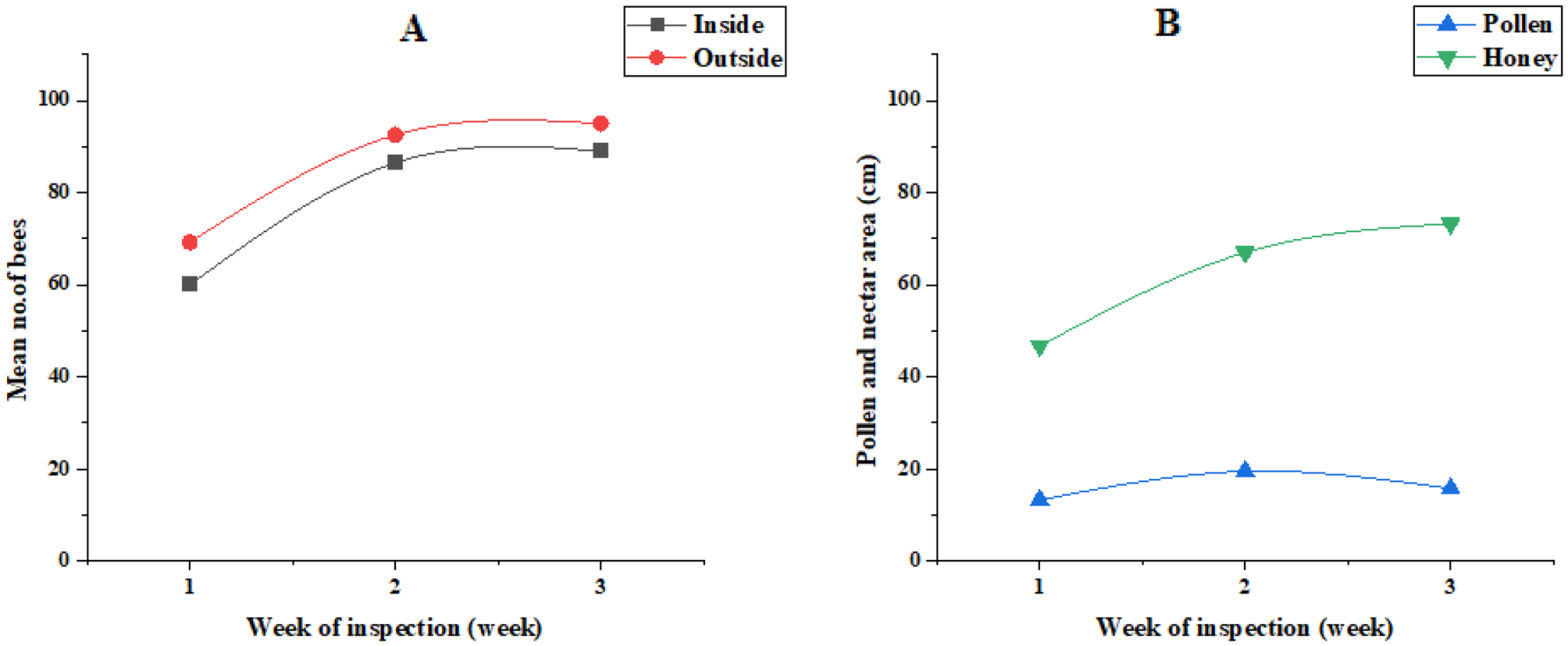
Relationship between fennel plant and bee foraging behavior (**A**), and food storage (**B**).

Foraging outside the hive (Out): The data indicates that fennel plant exposure has a positive impact on the number of *honeybee (A. mellifera)* engaged in foraging outside the hive. Over the three-week period, the number of *honeybee (A. mellifera)* observed foraging outside the hive increased from 69.25 to 95.00 on average (Fig. 2). The R squared value (0.8811) indicates that approximately 88.11% of the variability in this foraging behavior can be attributed to fennel exposure. The extremely low p-value (< 0.0001) indicates a strong statistical significance for this effect.

Activities inside the hive (In): Similarly, fennel exposure seems to positively influence the number of bees foraging inside the hive. The number of bees observed foraging inside increased from 60.00 to 89.00 over the study period (Fig. 2). The R squared value (0.8963) suggests that about 89.63% of the variability in this behavior can be explained by fennel exposure. The p-value (0.0001) highlights the statistical significance of this effect.

Pollen collection: The amount of pollen collected by the *honeybee (A. mellifera)* also appears to be influenced by fennel exposure. However, the R squared value (0.4958) suggests that fennel exposure explains about 49.58% of the variation in pollen collection, which is comparatively lower than the other parameters. The p-value (0.0459) is significant, but not as strongly as for other behaviors, indicating a moderate impact of fennel exposure on pollen collection.

Honey collection: Fennel exposure positively impacts the amount of honey collected by the bee colony. Honey collection increased from 46.75 to 73.25 over the three weeks. The R squared value (0.9016) suggests that approximately 90.16% of the variation in honey collection can be attributed to fennel exposure. The p-value (< 0.0001) signifies the strong statistical significance of this effect.

##### The impact of fennel plant colony activities

The presented Table 4 outlines the effects of fennel plant exposure on various parameters of bee colonies over 3 consecutive weeks.

**Table 4:** Impact of fennel plant exposure on bee colony parameters.

Seed nu for umbrellas	First week	Second week	Third week	R squared (eta squared)	p-value (95%)
Sealed brood	357.5 ± 19.84	557.5 ± 11.99	772.5 ± 25.29	0.9606	< 0.0001, 109.8
Unsealed broad	176.3 ± 12.48	265.0 ± 10.99	343.8 ± 23.75	0.8478	0.0002, 25.06
Pollen	53.25 ± 2.689	173.8 ± 6.25	257.5 ± 6.614	0.9874	0.0459, 4.351.2
Honey	257.5 ± 9.242	477.5 ± 12.50	877.5 ± 20.56	0.9900	446.0, < 0.0001
Colony strength	7.750 ± 0.25	9.00 ± 0.00	10 ± 0.00	0.9313	61, < 0.0001

**Figure 3:**
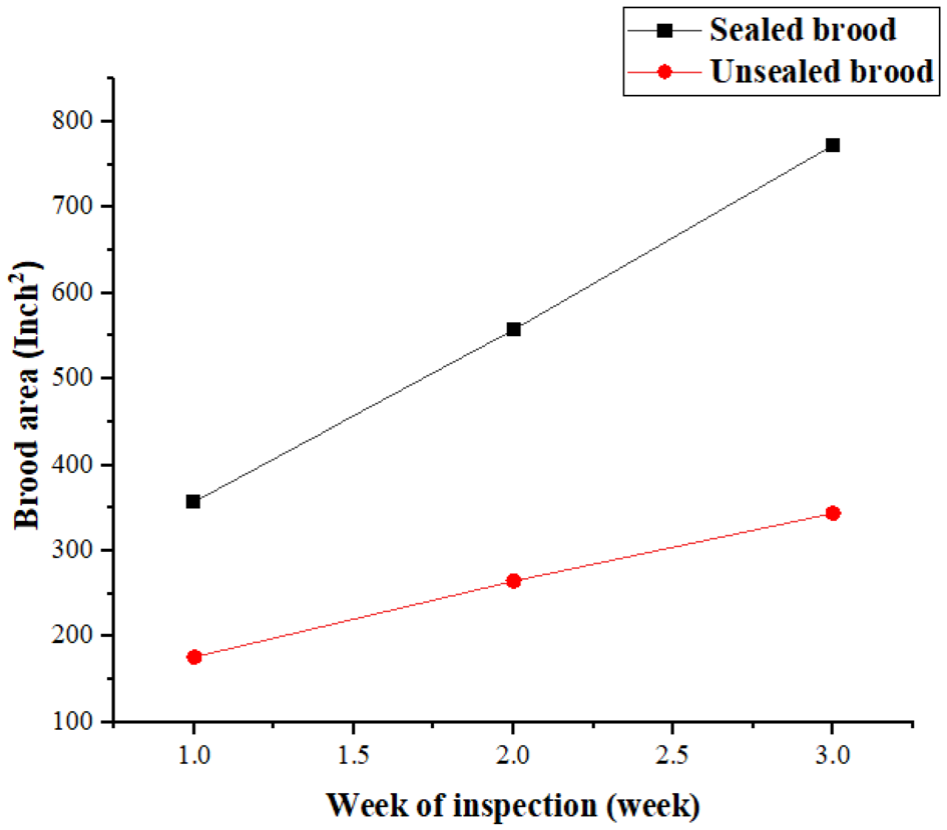
Effects of fennel plant on bee colony brood area (inch^2^).

Sealed brood: The sealed brood refers to the capped cells containing developing bee larvae. The data demonstrates a significant positive impact of fennel exposure on sealed brood amounts. Over the three-week period, the sealed brood amount increased from 357.5 to 772.5 cells on average (Fig. 3). The high R squared value (0.9606) indicates that about 96% of the variability in sealed brood amounts can be attributed to fennel exposure. The extremely low p-value (< 0.0001) further reinforces the statistical significance of this result.

Unsealed brood: Unsealed brood includes the bee larvae in open cells. Similar to sealed broods, fennel exposure positively influenced unsealed brood amounts. The values increased from 176.3 to 343.8 over the three weeks. Although the R squared value (0.8478) is slightly lower than for the sealed brood, it still suggests a strong relationship. The p-value (0.0002) confirms the statistical significance of the effect.

Pollen: Pollen plays a crucial role in bee nutrition. Fennel exposure has a substantial positive impact on pollen collection. The pollen amounts increased from 53.25 to 257.5 over the study period. The R squared value (0.9874) indicates that fennel exposure explains about 98.7% of the variability in pollen amounts. The p-value (0.0459) is below the conventional significance level of 0.05 but is higher than for other parameters, suggesting a strong effect that may be influenced by other factors.

**Figure 4:**
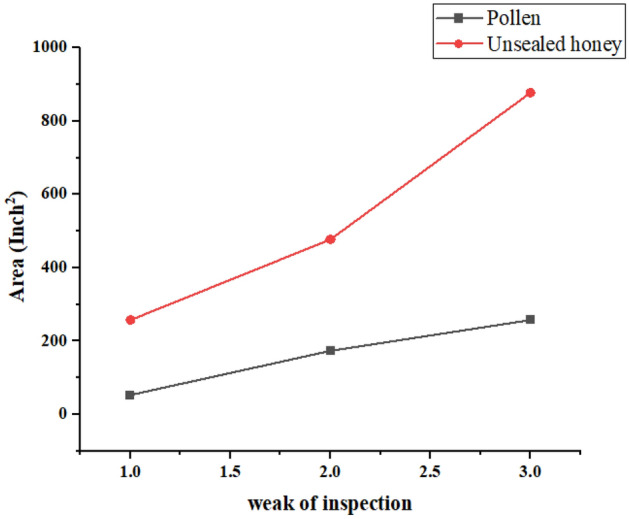
Increased honey accumulation in bee colonies with fennel: 3-week.

Honey: Honey is a vital energy source for bee colonies. The table shows a significant positive impact of fennel exposure on honey accumulation. Honey amounts increased from 257.5 to 877.5 over 3 weeks (Fig. 4). The high R squared value (0.9900) indicates that approximately 99% of the variation in honey amounts can be attributed to fennel exposure. The p-value (< 0.0001) underscores the robust statistical significance of this effect.

**Figure 5:**
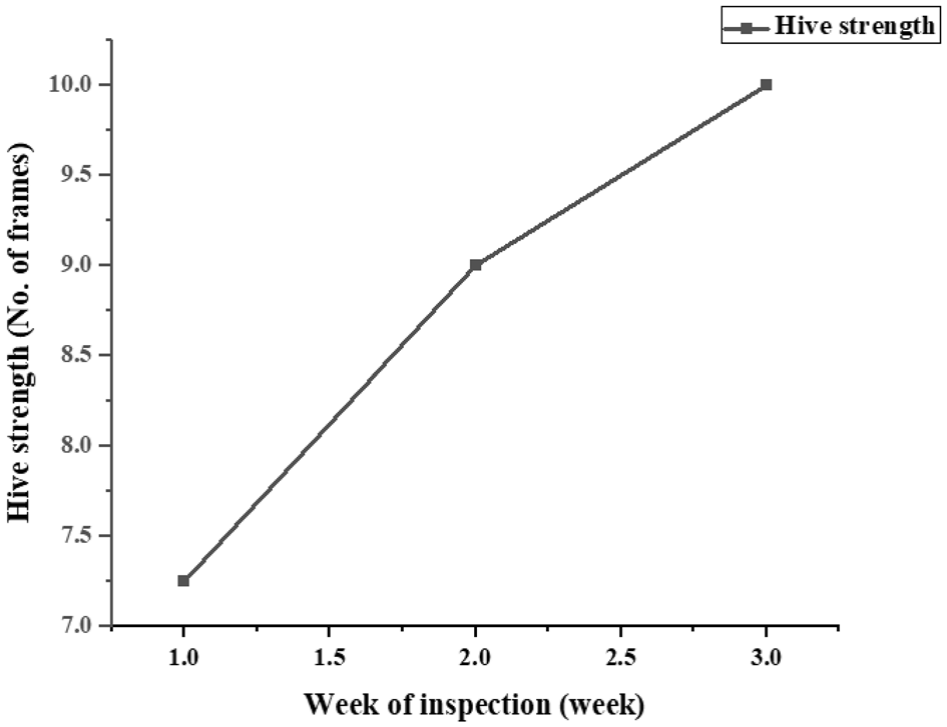
Enhanced colony strength with fennel exposure: impact over 3 weeks.

Colony strength: Colony strength is a measure of overall colony health and vigor. Fennel exposure is correlated with increased colony strength over the 3-week period. Colony strength values rose from 7.750 to 10 on average (Fig. 5). The R squared value (0.9313) indicates that about 93.1% of the variability in colony strength is explained by fennel exposure. The p-value (< 0.0001) reinforces the statistical significance of this effect.

### Sealed brood

The results revealed noteworthy variations among inspection periods in terms of sealed brood rearing by worker *honeybee (A. mellifera)* produced by colonies throughout the study period.

### Pollen and honey

### Strength

#### Impact of honeybee (*A. mellifera*) on fennel crop

##### Post-harvest quality, morphology, and commercial grades

**Figure 6:**
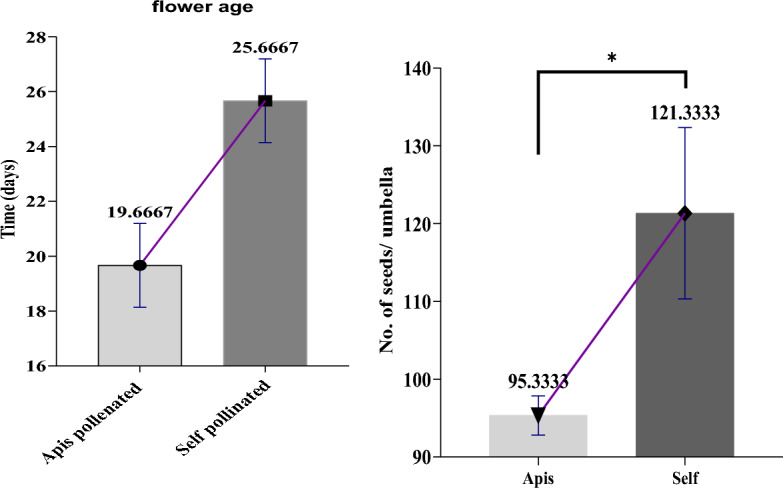
Comparison of fennel flower age and seed counts: apis vs. self-pollinated variants.

Flower age and number estimation of seed on umbrella: Table 5 reveals the impact of bee pollination on fennel flower age and seed number of umbrellas. Bee-pollinated flowers have a higher average age (25.67) compared to self-pollinated ones (19.67), with significant differences indicated by the low p-value (0.0086). Additionally, bee-pollinated flowers have more seeds per umbrella (121.3) than self-pollinated ones (95.33), with a significant p-value (0.0163). These findings underline the positive influence of bee pollination on fennel reproductive characteristics and potential plant productivity (Fig. 6).

**Table 5:** Estimations of fennel flower age, seed counts, and plant productivity.

Treatments	Flower age			Seed no. of umbrellas		
	Means	R squared	p-value (95%)	Mean	R squared	p-value
Self	25.67	0.853	0.0086	121.3	0.8	0.0163
Apis	19.67			95.33

Seeds size and weight: The unpaired t-test data revealed a significant difference between open-pollinated and self-pollinated treatments in terms of physical parameters of fennel seeds, i.e., seeds size (length) and seeds weight. Where bee pollination has a significant positive impact on important plant seed characteristics compared to self-pollination. The mean difference in seed length is 2.133 mm (± 0.558 SEM), and for seed weight, it is 0.273 g/100 seeds (± 0.045 SEM) between the two pollination treatments. The confidence intervals for both measurements indicate statistically significant differences between the treatments, as the confidence intervals do not overlap with zero, and the high R-squared values (0.343 for length and 0.906 for weight) suggest the pollination treatments explain most of the variation in seed characteristics. The p-values of 0.0007 (95% confidence) and 0.0034 (99% confidence) show highly significant differences between bee-pollination and self-pollination treatments. Bee-pollinated seeds tend to be longer and heavier.

Fruit weight per Umbel: In the realm of self-pollination treatment, the fennel fruit weight per umbel was recorded at 0.226 ± 0.0035 g (mean ± SD). Specifically, for the insect pollination treatments, the weight was measured at 0.619 ± 0.0324 g. In contrast, the treatments involving pollination without *honeybee (A. mellifera)* highlighted lower weights, with values of 0.226 ± 0.0035  g (Table 6).

**Table 6:** Comparison of seed characteristics between bee-pollinated and self-pollinated fennel seeds.

Pollination treatments	Length (mm)	Weight (g/100 seeds)	Fruit weight per umbel(g)
(Bee-pollination) A	6.533	0.510	0.619 ± 0.0324
(self-pollination) B	4.400	0.237	0.226 ± 0.0035
Difference between means (A − B) ± SEM	2.133 ± 0.558	0.273 ± .045	0.393 ± 0.0326
R squared (eta squared)	0.343	0.906	0.9733
p-value (95 and 99%)	0.0007	0.0034	0.0003

The statistical analysis showed significant differences. It highlighted dissimilarities between both fennel pollination treatments (Chi-square = 7.13, df = 1, p-value = 0.008) as well as variations among the treatments (Chi-square 0.9733, df = 2, p-value < 0.0003).

Our rigorous statistical analysis revealed a compelling and significant difference between grain weight when *honeybee (A. mellifera)* were present and grain weight in the absence of *honeybee (A. mellifera)* (p = 0.0034, two-tailed). The grain weight means for the presence of *honeybee (A. mellifera)* (0.5100) exceeded the mean for the absence of *honeybee (A. mellifera)* (0.2367), with a remarkable difference of 0.2733 ± 0.04410 (95% confidence interval 0.3958–0.1509).

#### Seeds morphology

##### Seeds colors

The coloration of fennel seeds varied according to pollination treatment. Figure 3 visually illustrates the stark disparities between seeds produced from plants pollinated by bees and those from non-bee-pollinated plants. Bee-pollinated fennel seeds exhibited more naturally intense light greenish-brown pigmentation compared to self-pollinated fennel, which appeared in a grayish-green color, as well as a blackish-brown color. Thus, bee pollination Fennel yielded seeds are typically brighter, more intensely light greenish brown compared to self-pollinated fennel-yielded seeds, which were darker and greenish brown.

##### Seeds shape colors

**Figure 7:**
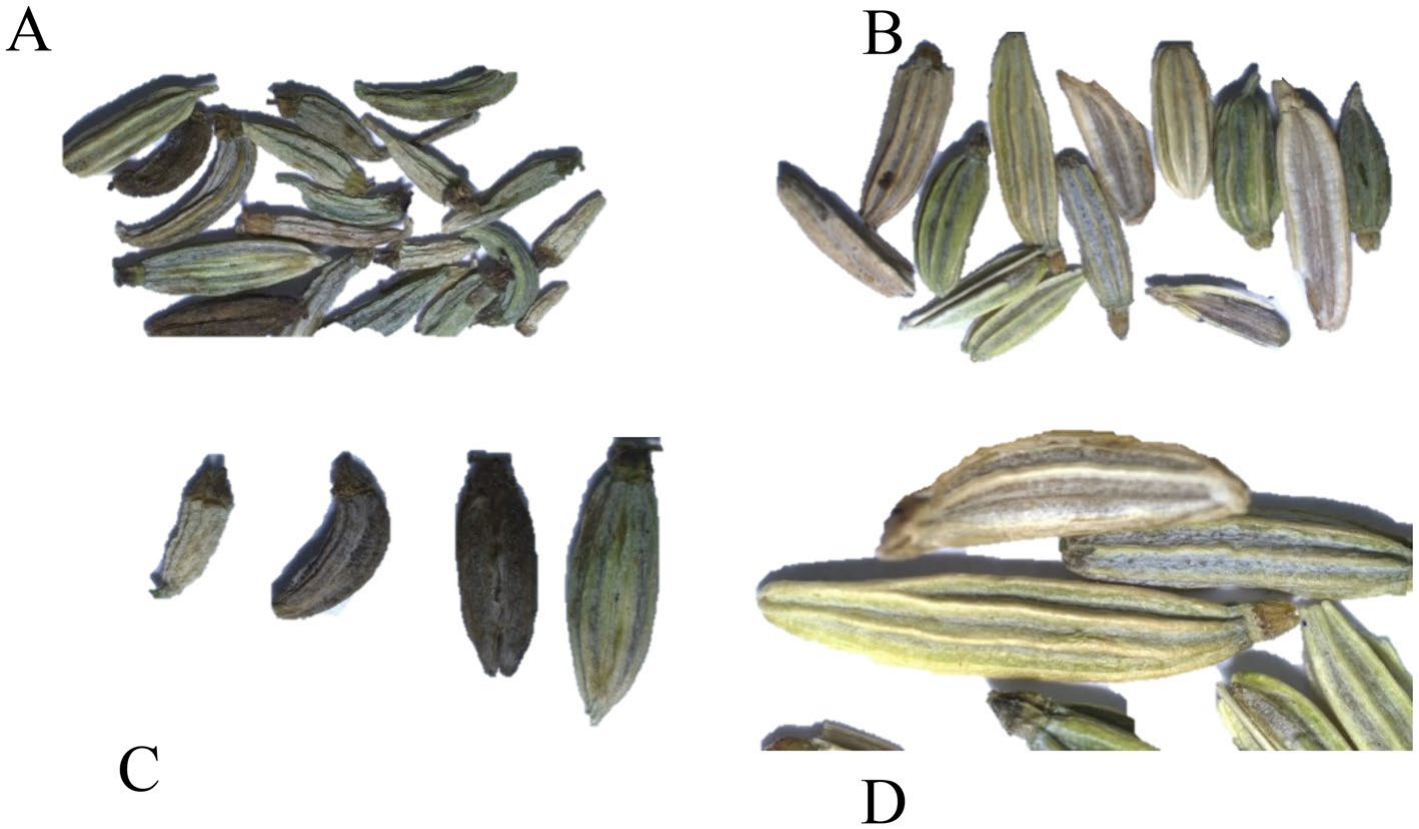
Differences in seed size and weight between bee-pollinated and self-pollinated fennel seeds.

The coloration of fennel seeds varied according to pollination treatment. Figure 7 visually illustrates the stark disparities between seeds produced from plants pollinated by bees and those from non-bee-pollinated plants. Bee-pollinated fennel seeds exhibited more naturally intense light greenish-brown pigmentation compared to self-pollinated fennel, which appeared in a grayish-green color, as well as a blackish-brown color. Thus, bee pollination Fennel yielded seeds are typically brighter, more intensely light greenish brown compared to self-pollinated fennel-yielded seeds, which were darker and greenish brown.

**Figure 8:**
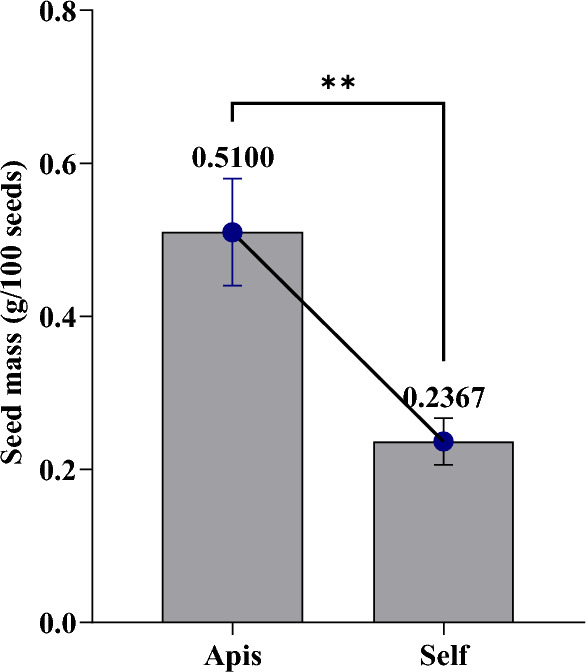
Differences in seed size and weight between bee-pollinated and self-pollinated fennel seeds.

Regarding shape, Seeds derived from bee pollination exhibited remarkable shape uniformity, demonstrating the consistent influence of this pollination method. In contrast, significant disparities were evident in the shapes of grains resulting from self-pollination in plants, indicating a distinct and notable effect of the pollination process (Fig. 8).

## Discussions

### Insect diversity and attraction by fennel

During the duration of our investigation, we documented a wide range of insect visitors to the blooming umbels of the fennel plant. This comprehensive examination revealed a diverse array of pollinators, including multiple bee species such as honeybees (Apis mellifera), carpenter bees, and parasitic wasps, as well as hoverflies and ladybug beetles. These findings align with research conducted by Layek et al.^26^, who reported a high diversity of floral visitors on fennel crops in India, highlighting the importance of Apis mellifera as a key pollinator. Similarly, Kumar and Singh^27^ elucidated the significant influence of insects on fennel crop pollination dynamics, emphasizing the pivotal role of honeybees. In Hisar, India, Shilpa et al.^10^ identified 25 insect species from five orders visiting fennel flowers, with bees such as *Apis florea*, *A. cerana indica*, *A. mellifera*, and *A. dorsata* being the most frequent visitors. Furthermore, Bharti et al.^9^ identified 25 insect species from 15 families and five orders as pollinators of fennel flowers, noting the high efficiency of *A. mellifera* as a pollinator.

Fennel flowers possess traits that are highly attractive to bees. These traits include abundant nectar and pollen rewards, bright clusters, and strong scents that guide bees efficiently. The shape of fennel flowers ensures effective pollen transfer during feeding, maximizing cross-pollination. Fennel blooms coincide with periods of high bee activity, providing ample resources when competition is low, making fennel an optimal choice for bees. These characteristics enhance resource availability and reproductive success for both fennel and its pollinator bees.

### Impact of fennel on bee activities

Bee foraging behavior is influenced by numerous factors, including environmental conditions, floral resources, and foraging strategies. Our study reveals variations in bee foraging on fennel flowers, particularly in umbel visitation frequency. We observed an increasing trend in bee activity over three weeks, indicating heightened foraging efficiency. *Apis mellifera* consistently demonstrated significant pollination prowess, making it a crucial contributor to the pollination process of fennel. The statistical analysis supports these findings, showing that fennel exposure significantly impacts bee foraging outside the hive, accounting for approximately 88.11% of the variability observed. Inside the hive, fennel exposure positively influences pollen collection and honey accumulation, with significant increases documented over the study period.

Several studies corroborate the impact of fennel on honeybee foraging behavior. For example, Carisio et al.^28^ emphasized the substantial influence of insect foraging on nectar productivity in flowers, suggesting shorter-term nectar volume measurements as a reliable assessment method. Layek et al.^26^ highlighted the high number of pollen grains carried by honeybees on their body surfaces, reinforcing the significant role of fennel in enhancing pollination efficiency.

### Impact of honeybee pollination on fennel

Our study underscores the pivotal role of honeybee (*Apis mellifera*) pollination in enhancing fennel (*Foeniculum vulgare*) crop productivity. By meticulously monitoring various parameters, we observed significant improvements in both the qualitative and quantitative aspects of fennel plants exposed to honeybee activity compared to those excluded from pollination.

Our primary observations indicated a substantial increase in seed set, seed weight, and overall crop yield in bee-pollinated fennel plants. Specifically, the seed set in these plants exhibited an approximate increase of 25%, and the seed weight showed significant enhancement. This improvement is attributed to the high pollen transfer efficiency facilitated by honeybees, which spend an average of 3.2 s per flower, ensuring effective pollination and fertilization rates.

In contrast, fennel plants isolated from honeybee pollination exhibited lower seed set and reduced seed weight, highlighting the crucial role of honeybees in enhancing crop productivity. Statistical analysis confirmed these findings, demonstrating a strong positive correlation (r = 0.85, p < 0.01) between honeybee visitation frequency and fennel seed yield.

Our findings align with and expand upon existing literature. Layek et al.^26^ reported that honeybees are primary pollinators for fennel crops in India, significantly contributing to improved crop yield. Their study noted a 20% increase in seed set due to honeybee pollination, closely mirroring our observed increase. Similarly, Kumar and Singh^27^ emphasized the critical role of honeybees in enhancing fennel seed quality and quantity, attributing these benefits to efficient pollination services provided by honeybees.

Shilpa et al.^10^ documented that fennel plants visited by honeybees showed a 22% increase in seed yield compared to those not visited by bees, aligning with our findings, and highlighting the consistency of honeybee pollination benefits across different geographic locations. Additionally, Bharti et al.^9^ identified *Apis mellifera* as the most effective pollinator among various insect species, supporting our observations regarding the superior pollination efficiency of honeybees.

### Ecological and agricultural implications

The ecological interaction between honeybees and fennel plants not only benefits crop productivity but also enhances environmental sustainability. Honeybees facilitate cross-pollination, contributing to genetic diversity and resilience in fennel populations. This mutualistic relationship ensures that fennel plants receive necessary pollination services to produce viable seeds, while honeybees benefit from the abundant nectar and pollen resources provided by fennel flowers.

Our study reinforces the importance of conserving honeybee populations and promoting practices that support pollinator health. Sustainable agricultural practices incorporating bee-friendly habitats can lead to increased crop yields and improved agricultural sustainability. By fostering an environment conducive to honeybee activity, farmers can enhance the productivity of fennel and other crops, ensuring long-term agricultural success.

## Conclusion

In our study, we found that various insects, including different bee species, visited fennel flowers for pollination. Notably, honeybees *Apis mellifera* was the most effective pollinators due to their frequent visits and high pollen loads. The abundant fennel blooms were a great additional food source for honeybees. Hives near fennel fields saw significant improvements in colony health, with more sealed brood, food storage, and overall brood production. Comparing bees' effect on fennel plots, honeybee visits boosted attributes like fruit set, weight, and seed production. These benefits underline the economic value of conserving wild bee populations that pollinate fennel. Our study shows how fennel and honeybees help each other. Fennel provides food for bees, and bee pollination enhances fennel growth. To sustainably produce fennel, we suggest managing both crops and pollinators. Future research can explore landscape management to optimize these mutual bee-crop relationships.

## Data Availability

The datasets generated during and/or analyzed during the current study are available from the corresponding author on reasonable request.
